# Single-Fiber Diffuse Reflectance Spectroscopy and Spatial Frequency Domain Imaging in Surgery Guidance: A Study on Optical Phantoms

**DOI:** 10.3390/ma14247502

**Published:** 2021-12-07

**Authors:** Polina S. Tseregorodtseva, Kirill E. Buiankin, Boris P. Yakimov, Armais A. Kamalov, Gleb S. Budylin, Evgeny A. Shirshin

**Affiliations:** 1 Faculty of Physics, M.V. Lomonosov Moscow State University, 119991 Moscow, Russia; tceregorodtceva.ps19@physics.msu.ru (P.S.T.); buiankin.ke17@physics.msu.ru (K.E.B.); bp.jakimov@physics.msu.ru (B.P.Y.); 2 Medical Research and Education Center, M.V. Lomonosov Moscow State University, 119991 Moscow, Russia; armais.kamalov@rambler.ru (A.A.K.); gleb.budylin@gmail.com (G.S.B.); 3 World-Class Research Center “Digital Biodesign and Personalized Healthcare”, Sechenov First Moscow State Medical University, 119048 Moscow, Russia; 4 Institute of Spectroscopy of the Russian Academy of Sciences, 108840 Moscow, Russia

**Keywords:** surgery guidance, diffuse reflectance spectroscopy, tumor detection, spatial frequency domain imaging (SFDI), optical phantoms

## Abstract

Diffuse reflectance spectroscopy (DRS) and imaging are increasingly being used in surgical guidance for tumor margin detection during endoscopic operations. However, the accuracy of the boundary detection with optical techniques may depend on the acquisition parameters, and its evaluation is in high demand. In this work, using optical phantoms with homogeneous and heterogeneous distribution of chromophores mimicking normal and pathological bladder tissues, the accuracy of tumor margin detection using single-fiber diffuse reflectance spectroscopy and spatial frequency domain imaging was evaluated. We also showed how the diffuse reflectance response obtained at different spatial frequencies with the spatial frequency domain imaging technique could be used not only to quantitatively map absorption and scattering coefficients of normal tissues and tumor-like heterogeneities but also to estimate the tumor depth localization. The demonstrated results could be helpful for proper analysis of the DRS data measured in vivo and for translation of optical techniques for tumor margin detection to clinics.

## 1. Introduction

Optical techniques are increasingly being used for intraoperative diagnostics to improve the sensitivity and specificity of tumor margin detection. Various optical methods, including diffuse reflectance spectroscopy, Raman and fluorescence spectroscopy with multiwavelength excitation of exogenous and endogenous tissue fluorophores [1,2,3,4,5], fluorescence lifetime imaging [6,7], optical coherence tomography [8,9], photoacoustics [10], terahertz spectroscopy [11,12], and other methods and their combinations are used to diagnose normal and abnormal tissue sites both ex vivo and in vivo.

The method used for real-time in vivo diagnostics should be robust and based on an easy-interpretable marker that allows separating healthy and pathological tissues while being fast and technically compatible with medical devices used during the surgery. DRS could be used to classify bladder cancer tumors using the significant difference in the vascularization of normal and tumor sites (i.e., based on the differences in the hemoglobin concentration) [13,14]. The diffusely reflected light could be captured using various imaging schemes; some of them, like narrow-band imaging, are already used in clinically approved cystoscopes [15,16]. Another possibility is using a single optical fiber inserted in the endoscope, which allows measuring the signal from a specific point of the tissue. The second modality, which is highly attractive for clinicians, are imaging techniques that allow for real-time visualization of tissue content. One of the prospective imaging techniques based on diffuse reflectance of light is the spatial frequency domain imaging (SFDI) method [17,18,19]. SFDI is a modern technique that allows one to quantitatively restore the absorption and scattering coefficients of the tissues [18], which is possible using narrow-band imaging or white light cystoscopy, where only semiquantitative or qualitative differences between colors of the tissues can be assessed. SFDI also allows performing the depth tomography, as the penetration depth of the structured light is dependent on the spatial frequency [20]. Moreover, SFDI can also be implemented as an endoscopic system [21,22].

A crucial step in understanding the possible pitfalls of the implemented technique is the analysis of its capabilities using simple objects with a priori known optical properties. For this purpose, materials that mimic the optical properties of real tissues can be used. One of the frequently used options is the use of silicones with built-in scatterers such as TiO_2_ and dyes as absorbers [23,24].

In this work, using PDMS-based optical phantoms with homogeneous and heterogeneous distributions of the chromophores mimicking optical properties of normal and pathological bladder cancer, we evaluated how the diffuse reflectance parameters and DRS-based tumor margin assessment obtained using the single-fiber scheme and SFDI depend on the detection parameters, e.g., on the position of the fiber relative to the tissue surface, and the properties of the chromophoric inhomogeneities. Comparative analysis of point measurements in a single-fiber scheme and of the SFDI-based imaging scheme allowed demonstration of the advantages and drawbacks of these methods in guided surgery. The obtained results are essential for a proper analysis of the DRS data measured in vivo and for the translation of optical techniques for tumor margin detection to clinics.

## 2. Materials and Methods

### 2.1. Fabrication Protocol of Homogeneous PDMS Optical Phantoms

To evaluate the applicability of diffuse reflectance spectroscopy and imaging for intraoperative diagnostics of tumor margins implemented as a single-fiber scheme and SFDI-scheme, the optical phantoms that simulate the properties of tissues were prepared. Polydimethylsiloxane (PDMS) (Sylagerm-2106, Lyubertsy, Russia) was used as a matrix containing the scattering particles of TiO_2_ (RusChem, Saint-Petersburg, Russia) and the acrylic and alcohol dyes with characteristic absorption peaks in the 500–600 nm range, which determined the absorption coefficient of the phantoms. The reduced scattering coefficient of the TiO_2_ particles was characterized using the SFDI method. The fabrication method utilized in this work is based on the protocol previously described in [25].

The final concentration of TiO_2_ particles and the dye in the homogeneous optical phantoms, studied in Section 3.1 of the Results, corresponds to reduced scattering coefficient of μ_s_’ = 120 mm^−1^ and absorption coefficient μ_a_ = 0.03 mm^−1^ at 550 nm wavelength, in agreement with scattering and absorption coefficients of normal bladder tissue reported in [24]. The tumor phantom had the same scattering coefficient and the increased absorption coefficient μ_a_ = 0.06 mm^−1^, in agreement with the properties of vascularized tumor regions of the bladder [26]. The phantoms had a cylindrical shape with a diameter of 35 mm and a thickness of 5 mm.

### 2.2. Fabrication of a Non-Uniform Phantom to Determine the Lateral Accuracy of a Single-Fiber Scheme

The optical phantom used to assess the accuracy of tumor localization using a single-fiber scheme was manufactured in two stages. First, a base phantom, simulating the optical properties of healthy tissue containing special protrusions, was prepared. After the base phantom had cured, a mixture simulating the properties of a tumor was poured into the protrusions and then cured. The diameter and the depth of inhomogeneity were 9 mm and 1.5 mm, respectively.

### 2.3. Production of an Inhomogeneous Optical Phantom with a Varying Thickness of Inhomogeneity

This phantom was also produced in two steps. The first part of the phantom mimicked the tumor tissue of the bladder and had the form of a truncated cylinder. It was placed on the bottom of the mold, which was then filled with the PDMS-mixture, mimicking the optical properties of the normal tissue, which was then cured.

The final concentration of titanium dioxide in both parts of the phantom corresponded to µ_s_’ (550 nm)~1.3 mm^−1^; the concentration of the alcohol ink in phantoms of healthy and tumor tissue corresponded to absorption coefficients µ_a_ = 0.1 mm^−1^ and 0.2 mm^−1^ at 550 nm, respectively.

### 2.4. Single-Fiber Measurement of Diffuse Reflectance Spectra

**Figure 1:**
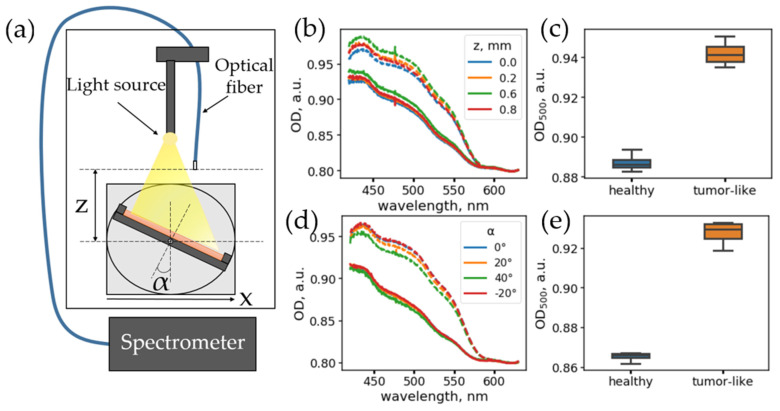
(**a**) Scheme of a single-fiber setup for measuring reflected light spectra. (**b**) Dependence of optical density (OD) spectra on the fiber position above the surface, *z*, for phantoms of normal tissue (solid lines) and tumor tissue (dashed lines). (**c**) Boxplots for optical densities of phantoms of normal (blue) and tumor (orange) tissue. Each box represents all the spectra measured for different positions, z. (**d**) Dependence of the OD spectral shape on the angle, α, between the fiber and the phantom surface. Solid lines correspond to the spectra of normal tissue; dashed lines correspond to tumor tissue phantom. (**e**) Boxplots for optical densities of phantoms of normal (blue) and tumor (orange) tissues. Each box represents all the spectra measured for different angles α.

To test the single-fiber scheme, imitating the conditions of an optical scheme implemented in an endoscope during surgery, a special experimental setup was created where the diffuse reflection spectra were recorded using an optical fiber under homogeneous illumination with an external source (Figure 1a).

A 5 W halogen lamp with a continuous spectrum in the range of 400–2500 nm was used as a light source. A multimode optical fiber with a core diameter of 550 μm and a numerical aperture of 0.27, connected to an Ocean Optics Maya 2000 Pro spectrometer(Orlando, FL, USA) records reflection spectra in the range of 200–1100 nm, was used as a detector. The optical fiber was connected to the holder moving up or down, while the optical phantom was placed on a platform (Figure 1a), allowing it to rotate the phantom and move the phantom perpendicular to the fiber.

The diffuse reflectance spectra were calculated from the spectra of the recorded intensity, *I*, as follows. For a switched-off lamp in the absence of external illumination, the spectrum of the background noise of the detector *I_bg_* was measured, followed by the intensity of the signal reflected off the phantom.

For each recorded intensity spectrum, *I*, the reflectance spectrum, *R*, was calculated as:

(1) $$R=\frac{I-I_{bg}}{I_{ref}-I_{bg}},$$

where *I_bg_* is the background noise of the detector and *I_ref_* is the reference spectra obtained for the Spectralon sample located at 2 mm from the fiber end.

The effective optical density of the sample was calculated as:

(2) OD=−ln(R).

### 2.5. Spatial Frequency Domain Imaging: Experimental Setup and Analysis Algorithm

A separate custom-build setup was used to implement the spatial frequency domain-imaging method. A commercially available digital micromirror device (DMD)-based projector (TouYinger S7, Everycom Technology, Shenzhen, China) was used to project patterns. The LED light sources of the projector were replaced with a 35 W incandescent halogen lamp.

To detect the signal, a monochrome CMOS camera (CS135MUN, Thorlabs Inc., Newton, NJ, USA) was used. The images with 1024 × 1280 pixels were captured using a varifocal objective (20R0001604, Navitar, Rochester, NY, USA). Dichroic bandpass filter, passing the light in the 550 ± 20 nm spectral range, was used for the spectral selection.

The projector illuminated the surface of the optical phantoms with intensity patterns modulated with a sine function with given spatial frequencies *f_x_*, varied in the range from 0 ${\mathrm{mm}}^{-1}$ (constant illumination) up to 0.2 ${\mathrm{mm}}^{-1}$ with the step of ~0.013 ${\mathrm{mm}}^{-1}$, according to Equation (3):

(3) $$I\left(x\right)=I_{0}\;\left(1+M\sin\left(2\pi f_{x}x+\alpha \right)\right)$$

with a modulation depth *M* ~0.95. The obtained raw maps of the intensity patterns projected onto the phantom’s surface were processed to obtain diffuse reflectance maps at different spatial frequencies and absorption and scattering coefficients using the algorithm presented in [18]. We briefly point out that the response from the tissues for each pixel, x, of an image, $M_{AC}\left(x,f_{x}\right)$, for the specific spatial frequency was calculated using the intensity maps obtained for three phase shifts of the projected pattern: $\alpha_{1}=0,\;\alpha_{2}=2/3\pi ,\;$and $\alpha_{3}=4/3\pi$, according to Equation (4):

(4) $$M_{AC}\left(x,f_{x}\;\right)=\frac{2^{\frac{1}{2}}}{3}\;\{{\left[I_{1}\left(x\right)-I_{2}\left(x\right)\right]}^{2}+{\left[I_{1}\left(x\right)-I_{3}\left(x\right)\right]}^{2}+{\left\{{\left[I_{2}\left(x\right)-I_{3}\left(x\right)\right]}^{2}\right\}}^{\frac{1}{2}},$$

where $I_{1},I_{2},I_{3}\;$represent intensity, I(x), values projected with different phase shifts ($\alpha_{1},\;\alpha_{2}$, and $\alpha_{3}$, respectively). The diffuse reflection at zero frequency,$\;f_{x}=0\;{\mathrm{mm}}^{-1}$, was calculated according to Equation (5):

(5) $$M_{AC}\left(x,f_{x}=0\;{\mathrm{mm}}^{-1}\right)=\frac{1}{3}\left[I_{1}\left(x\right)+I_{2}\left(x\right)+I_{3}\left(x\right)\right]-I_{DC\;background}\left(x\right),$$

where $I_{DC\;background}\left(x\right)$ is the noise of the detector obtained when the light source was turned off. Using the detector response function, estimated by measuring liquid optical phantom with known optical properties, the diffuse reflectance coefficients $\left(R_{d}\right)$ of the heterogeneous optical phantoms at different spatial frequencies were restored and fitted to quantify absorption (μ_a_) and reduced scattering (μ_s_’) coefficients.

To calibrate the system, the liquid homogeneous phantom was used as a reference, in which a 20% lipofundin solution (Lipofundin MCT/LCT 20%, BBraun Melsungen AG, Melsungen, Germany) in water was used as a scatterer, and water-soluble nigrosine (Vektone, Saint Petersburg, Russia) was used as an absorber, so that the reduced scattering and absorption coefficients of reference phantom at 550 nm were 1.65 and 0.18 mm^−1^, respectively.

All spectra and image analysis was performed using custom-written scripts using Python3 (v 3.10, 2021, Python Software Foundation, Beaverton, OR, USA) and Scipy (v 1.7.1), Scikit-Image (v 0.18.3), Numpy (v 1.21.1), and LmFit (v 1.0.2) libraries.

## 3. Results

### 3.1. Diffuse Reflectance Spectra Are Weakly Dependent on Angle and Distance to Tissues

In endoscopic surgery, DRS can be implemented using a single detection fiber and wide-angle illumination from an endoscope. In general, a surgeon cannot precisely determine the position and angle of the fiber relative to the tissues. Hence, two major questions when using the single-fiber DRS scheme are: (1) Does the spectral band shape significantly depend on the angle of inclination of the fiber and the distance from the fiber end to the tissue surface? (2) How does the precision of tumor margin determination depend on the distance between the fiber and the surface? To answer these questions, we measured the diffuse reflectance spectra from the optical phantoms of various configurations using an experimental setup simulating the measurement conditions with an endoscope (Figure 1a, see Section 2.4 for details). The fiber was fixed above the optical phantom, located on a special platform allowing rotating and translating the phantom and changing the distance between the edge of the fiber and the phantom surface.

It should be noted that, in the case of a homogeneous sample and homogeneous incoherent illumination, there should be no dependence on the angle or distance to the object. However, this can be violated in the case of sample inhomogeneities, and this can be the case for in vivo measurements. Thus, to estimate the setup performance in the homogeneous case, the dependence of the diffuse reflectance spectra of optical phantoms on the angle and distance to the surface of homogeneous optical phantoms was investigated. A detailed description of the phantom preparation is presented in Section 2.1. Two phantoms imitating healthy tissue and tumor with increased vascularization were prepared, with absorption and scattering coefficients similar to the absorption and scattering coefficients of the bladder tissues in the spectral range of 500–650 nm. Their observed inhomogeneity of optical density, estimated as relative error for measurements at different points, was 0.5–0.6%.

We investigated how the diffuse reflectance spectra change as a function of the distance between the fiber and the surface of the optical phantoms when the fiber is located normally to the sample surface (α = 0°). For each configuration, the optical density (OD) spectrum was calculated as OD = −ln(*R*), where *R* is the diffuse reflectance spectrum calculated according to the procedure described in Section 2.3. The spectral region, 450–550 nm, of these spectra corresponds to the dye absorption region and can be used for its concentration estimation, while the 600–800 nm range can be used for baseline estimation. For both types of phantoms, the shape of the OD spectra does not depend on the distance to the fiber (Figure 1b). The variation in the OD values at a wavelength of 500 nm with a change in the distance, z, from the fiber to the phantom when varying from 0.1 to 0.8 mm was 0.3–0.7%, which corresponds to the phantom inhomogeneity level and is 2.8 times lower than the difference in effective optical density for the phantoms of normal and tumor tissue (Figure 1c).

Secondly, the dependence of the diffuse reflectance spectra on the orientation of the fiber relative to the surface was investigated. For this, the fiber was fixed at a distance of z = 2.0 mm from the phantom, and then the angle of inclination of the phantom, α, was varied from −40° to +40° with a step of 20°. Figure 1d shows the diffuse reflectance spectra for a phantom that mimics normal and tumor tissues for different fiber orientations. It can be seen that the spectra have a small variation at the 0.3–0.7% level, which corresponds to the inhomogeneity level, and is approximately three times lower than the differences observed for the phantoms of healthy and tumor tissues (Figure 1e).

The obtained results indicate that, in realistic conditions, in the case of increased tumor vascularization, the single-fiber scheme is suitable for detecting tumor tissue areas, while the detection can be carried out at different angles of fiber inclination. This fact is of practical value because rigid cystoscopy is often in use, and there is no possibility for the surgical fiber to bend. At the same time, the insignificant dependence of the reflection coefficients on the orientation of the fiber suggests that, using a single-fiber scheme, it is possible to detect hemoglobin variations three times lower than those observed in a real tumor, making it possible to detect earlier stages of increased vascularization and tumor development.

However, it is obvious that, because the optical fiber has a nonzero numerical aperture, with an increase in the distance between the fiber and the tissue under study, the accuracy of determining the tumor margin will gradually decrease. Below, we investigated how the spatial resolution of a tumor margin for the single-fiber scheme depends on the distance between the fiber and a non-uniform optical phantom.

### 3.2. The Accuracy of Tumor Margin Assessment Depends on the Fiber-Tissue Distance

**Figure 2:**
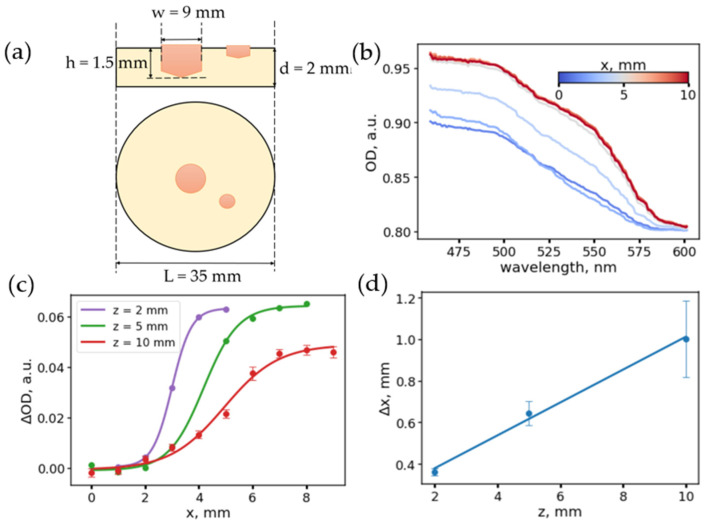
(**a**) Scheme of a heterogeneous phantom simulating a tumor. (**b**) Examples of the optical density spectra measured in different points of the phantom with fiber to tissue distance z = 2 mm. Variations in the spectrum band shape and the transition region are visible. (**c**) Dependence of the difference in absorption at wavelengths of 500 and 600 nm for different distances between the fiber tip and the phantom surface. (**d**) Dependence of the width of the transition zone on the distance between the surface and the fiber tip.

To assess how the accuracy of assessment of the tumor margin depends on the distance between the scanning fiber and the tissue surface, the following experiment was performed with a heterogeneous phantom containing two areas with optical properties of healthy and tumor tissue (Figure 2a). The optical parameters of the regions of this phantom correspond to the phantoms of healthy and tumor tissues described in the previous section and Section 2.2.

The diffuse reflection spectra were measured as the fiber moved along the phantom surface, while the fiber was oriented perpendicular to the sample surface. A series of longitudinal scans (along the *x* coordinate) were measured for three distances between the fiber and the phantom, *z*, of 2, 5, and 10 mm. Optical density spectra for scanning at a distance of z = 2 mm, depending on the lateral position of the fiber, x, are shown in Figure 2b. During the change of the fiber position from normal tissue to pathological, an increase in absorption in the range of 500–600 nm was observed due to an increased absorption coefficient of tumor tissues.

Thus, the OD at the wavelength 500 nm was chosen as an indicator that determines the transition between normal tissue and tumor. The dependencies of the ΔOD(*x*) = OD(500 nm) − OD(600 nm) on the position of the fiber, *x*, for various distances from the fiber tip to the phantom surface, *z*, obtained upon scanning, are shown in Figure 2c. As can be seen, as the distance from the fiber to the phantom decreases, the ΔOD(*x*) curves become steeper, while the amplitude of the ΔOD increases with decreasing distance. The error in determining the margin was estimated by fitting the ΔOD(*x*) dependencies to a sigmoid (Equation (6)).

(6) $$\Delta \mathrm{OD}\left(x\right)\sim \frac{A}{1+exp\left(-\frac{x-x_{0}}{\Delta x}\right)}$$

It was found that the parameter that determines the smoothness of the transition and, accordingly, the error, Δ*x*, of determining the transition boundary increases linearly with an increase in the distance between the fiber and the phantom surface (Figure 2d). This dependence can be explained by the fact that, with an increase in the distance between the fiber and the phantom, the effective light collection area increases and reflection both from the tumor and healthy tissue is captured. It can be concluded that, for a given fiber configuration, a scanning height of up to 10 mm makes it possible to determine the tumor border with an accuracy of up to 2 mm. The accuracy for the minimum height can be estimated as 0.36 mm (Figure 2d), which is close to the radius of the optical fiber of 0.275 mm.

### 3.3. Single-Fiber DRS Is Sensitive to Depth-Location of Tumor-like Chromophoric Inhomogeneities

**Figure 3:**
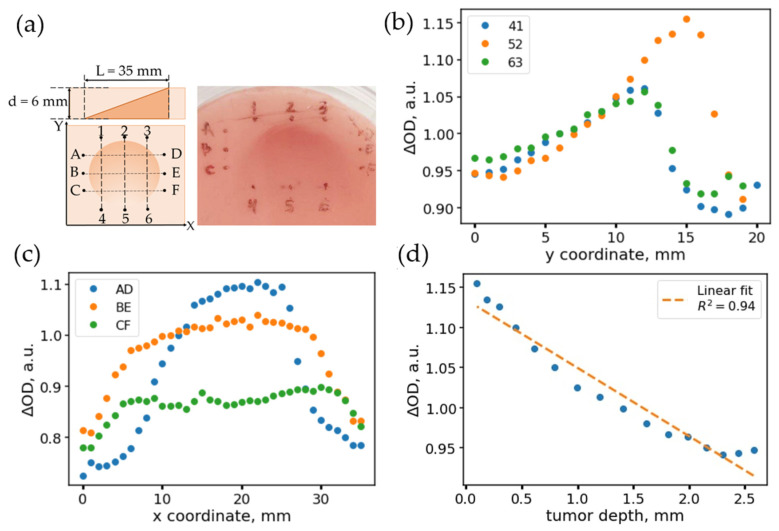
(**a**) Inhomogeneous phantom: cross-sectional view, scanning grid (top view), and phantom photo. (**b**) Dependences of the observed absorption coefficients along the scanning directions 41 (blue dots), 52 (yellow), 63 (green). The scans correspond to the direction along the varying thickness of the inhomogeneity, which corresponds to the changing values of the absorption coefficient. (**c**) Dependences of the observed attenuation coefficients along the scanning directions AD (blue points), BE (yellow), CF (green). The scans correspond to the direction along the constant thickness of the inhomogeneity, which corresponds to the plateaus in the central region of the scan. (**d**) Dependence of the absorption coefficient on the thickness of the inhomogeneity along the scan 52.

When determining the boundaries of various types of inhomogeneities, in particular, of a tumor, it can not only be non-uniformly distributed over the surface but also non-uniformly distributed with depth. To evaluate how the DRS signal varies in the case of chromophores that are inhomogeneously distributed with depth, we prepared an optical phantom of a special configuration. A detailed description of the preparation of this phantom is presented in Section 2.3. The phantom had the shape of a cylinder with a built-in inhomogeneity, which had the shape of a truncated cylinder with an increased value of the absorption coefficient (Figure 3a). This configuration of the phantom was necessary to create a smooth gradient of the inhomogeneity depth. The absorption coefficient of the surrounding “normal” tissue was 0.1 mm^−1^, and the absorption coefficient of inhomogeneity was 0.2 mm^−1^, while the scattering coefficients were similar and equal to 1.3 mm^−1^.

To assess the dependence of the measured parameters on the depth of the second layer with a single-fiber scheme, a coordinate grid was marked on the phantom surface and three scans were made along the direction of thickness change (scans “14”, “25”, “36”) and three transverse scans, along which the depth of the inhomogeneity remained approximately the same (scans “AD”, “BE”, “CF”). Figure 3b,c show the profiles of the value of ΔOD estimated as the difference between OD values at 550 and 600 nm, i.e., ΔOD = OD(550 nm) − OD(600 nm), obtained when scanning with a single-fiber scheme along the direction of change in the depth of heterogeneity location (Figure 3b) and along the lines of constant depth (Figure 3c). As can be seen, for scan 52, for which the thickness variation is the largest, ΔOD changes the most. In this case, in the region of 5–16 mm, and ΔOD increases linearly, corresponding to a linear decrease in the thickness of the phantom. In this case, the ΔOD profiles measured along the levels of constant depth of inhomogeneity demonstrate a “stepwise” dependence on the scanning coordinate X.

It should be noted that the steepness of the boundaries does not significantly depend on the depth of inhomogeneity. Thus, the accuracy of the inhomogeneity boundary determination is affected by the distance between the fiber and the surface of the tissue but not by the inhomogeneity depth.

As it can be seen from Figure 3c, the ΔOD value depends on the thickness of the phantom inhomogeneity. To correlate ΔOD with the true depth of the inhomogeneity, the phantom was later cut along the scan lines, the images were taken, and the depth of the inhomogeneity (distance to the measurement surface) was measured using ImageJ (v1.53m, 2021, Public domain). The correlation of the relative optical density with the thickness is presented in Figure 3d; ΔOD decreases linearly with increasing depth. From the deviation of the points from the linear relationship, it is possible to estimate the minimum inhomogeneity thickness that is possible to detect. The linear fit had a correlation coefficient of r = −0.971; the standard deviation of the values from the fitting curve was 0.0172. The slope coefficient of the straight line was −0.085 mm^−1^; thus, the minimum detectable thickness of inhomogeneity located on the surface can be estimated as ~0.2 mm.

### 3.4. Structured-Light Imaging Provides the Absorption and Scattering Coefficients and Depth Localization of Tumor-Like Inhomogeneities

As shown above, the single-fiber scheme allows localizing the position of the tumor laterally with reasonable accuracy. However, this detection scheme has several drawbacks. First, it is necessary to scan along the sample to localize the border in a larger field of view, while for accurate localization, it is necessary to perform scanning holding the fiber close to the tissue surface. Secondly, scanning with a single fiber, when the distance between the source and the detector is fixed, does not allow assessing the depth of the tumor without a priori knowledge of the optical density of the tumor and adjacent healthy tissue—the contrast of the absorption coefficient is directly correlated with the heterogeneity depth (Figure 3d).

**Figure 4:**
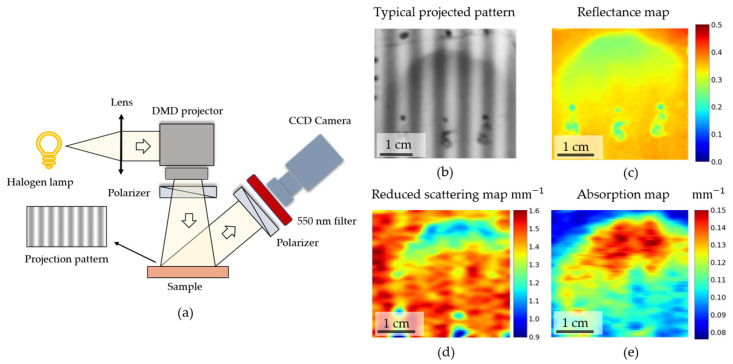
(**a**) Scheme of the experimental setup used to perform spatial frequency domain imaging. (**b**) An example of a raw intensity map obtained with structured illumination (spatial frequency f = 0.15 mm^−1^). (**c**) An example of a reflectance coefficient obtained after imaging (f = 0 mm^−1^). (**d**,**e**) Maps of reduced scattering (**d**) and absorption (**e**) coefficients obtained for inhomogeneous optical phantom using fitting of diffuse reflectance coefficients on frequency, as described in Section 2.5.

To eliminate these disadvantages, imaging methods should be used. One of the most recent diffuse imaging techniques is the spatial frequency domain imaging (SFDI) technique. The essence of the method is rigorously described in [18,19]. Several patterns with intensity varying along one of the spatial coordinates are projected onto the investigated area of the object and detected with a camera (Figure 4a). Detection of reflected light from a spatially modulated source and subsequent processing of the obtained images allows one to quantify the absorption and scattering coefficients in the case of homogeneous media, as well as to estimate the depth of inhomogeneities [18,20]. This assessment is possible because projecting and imaging patterns with different spatial frequencies of bright and dark stripes of light is analogous to measuring the signal for different distances between the source (“bright stripes”) and the detector (“dark stripes”), thus achieving detecting of light traveling at different trajectories and mean propagation depths by using different spatial frequencies of the projected pattern [19]. At the same time, the SFDI method can also be implemented as an endoscopic scheme [21], thus being suitable for intraoperative diagnostics during surgery on internal organs.

In our work, the ability to localize inhomogeneities and their localization depth, as well as the dependence of the accuracy of determining tumor localization for the SFDI method, were assessed. A detailed description of the experimental setup is presented in the Materials and Methods section (Section 2.5). Briefly, the setup included a DMD-based projector with a broadband (400–2500 nm) lamp capable of projecting patterns with spatial frequency down to 2.5 mm^−1^. Detection was carried out using a charge-coupled device (CCD) camera with a short-focus objective and a 550 ± 20 nm dichroic bandpass filter located in front of it. The setup is shown in Figure 4a.

Diffuse reflection maps were measured for spatial frequencies varying from f = 0 mm^−1^ up to f = 0.2 mm^−1^, with a step of f = 0.013 mm^−1^ for an optical phantom with inhomogeneity located at different depths, previously measured using a single-fiber setup (Section 2.4, Figure 3). Examples of the projection of spatially modulated light onto a phantom and the resulting diffuse reflection map are shown in Figure 4b,c. Using the diffusion approximation of light transfer in tissues and data for reference homogeneous liquid phantoms, the absorption and reduced scattering coefficients were estimated for the phantoms under the assumption of a homogeneous medium and diffuse approximation. The maps of the absorption and scattering coefficients are shown in Figure 4d,e. As can be seen, the absolute values of the absorption coefficients do not completely coincide with the real values of the absorption and scattering coefficients of the heterogeneity and the phantom that simulates the surrounding tissues. Such differences can be explained by the fact that the model used for processing does not take into account heterogeneously distributed tissues; therefore, the chromophore distribution is “averaged” over depth, and the estimated absorption coefficient depends on the depth distribution of the chromophore.

When processing the SFDI data under the assumption of homogeneously distributed chromophores, we found that the observed absorption coefficient is correlated with the depth of the heterogeneity in the phantom. This is due to the fact that the reflection coefficients for different spatial frequencies effectively collect the signal from different depths of the object under investigation [18,19,20].

**Figure 5:**
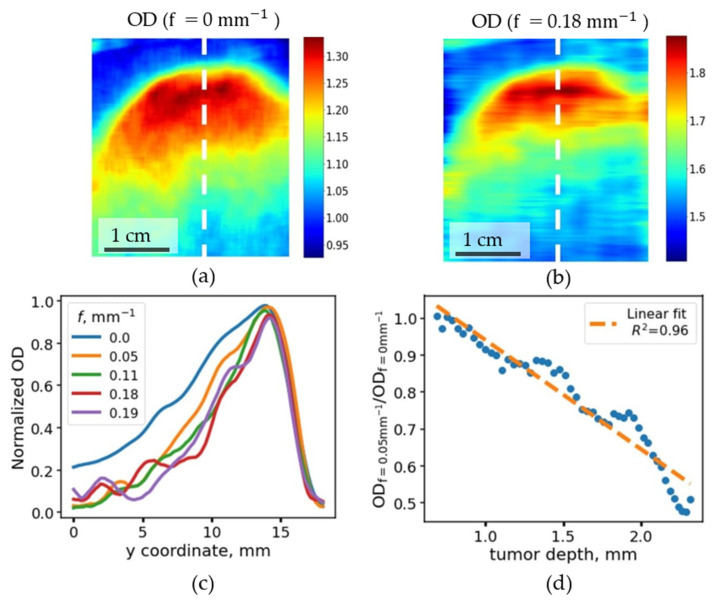
(**a**,**b**) Maps of optical density (OD) of inhomogeneous phantom, calculated as −ln(*R*), obtained at different spatial frequencies (f = 0 mm^−1^, (**a**)) and (f = 0.18 mm^−1^, (**b**)). The rapid decrease of the OD values at the higher spatial frequency, where an increase of inhomogeneity localization depth in panel (**b**) is observed. (**c**) Optical density, calculated along the y coordinate, corresponds to the change of the localization depth of the inhomogeneity normalized to the minimum and maximum values of the OD along the profile. (**d**) Correlation between the ratio of normalized optical depths at spatial frequencies of projected patterns f = 0.05 mm^−1^ and f = 0.0 mm^−1^ with the true localization depth of inhomogeneity.

It was also observed that the margins of inhomogeneities deeply located in the phantom show more contrast when measuring the diffuse reflection signal at low spatial frequencies, while the contrast at larger depths gradually decreases when increasing the modulation frequency (Figure 5b,c). This phenomenon can be explained by the fact that higher spatial frequencies correspond to a small distance between the source and the detector, thus at higher frequencies, only the photons from lower depths are detected.

Indeed, the profile of the normalized optical density, calculated as −ln(*R*), obtained at different spatial frequencies along the “25” profile has a maximum in the region of y = 15 mm, corresponding to the region where the inhomogeneity protruded onto the phantom surface (Figure 5c). It can be seen that the relative contrast in OD decreases in the left shoulder in Figure 5c, in the areas corresponding to a tumor located deep in the tissue. It can be seen that the OD at y = 6 mm normalized to the maximum value of OD (at y = 15 mm), gradually decreases from 0.4 for the f = 0 mm^−1^ down to 0.21 for frequencies above 0.15 mm^−1^. The lateral coordinate y = 6 mm corresponds to the depth of the chromophoric heterogeneity of 2 mm.

This fact was taken into account to create a semi-quantitative model to estimate the depth of the heterogeneity. The ratio of the optical density coefficients for different spatial frequencies was calculated, and it was correlated with the actual depth of the inhomogeneity, determined from the phantom cut along trajectory “25”. It was found that the ratio of the reflection coefficients positively correlates with the depth of occurrence of the inhomogeneity in the phantom (Figure 5d). This estimation demonstrates the applicability of the SFDI method in the assessment of depth distribution of chromophores in tissues.

## 4. Discussion

The obtained results show that the diffuse reflectance spectra obtained via the single-fiber scheme (Figure 1) vary insignificantly upon changing the fiber position relative to the tissue surface, and the changes of the optical density index are sufficiently lower upon changing the position of the fiber in comparison with the variation of the optical density, associated with the difference in concentration of the hemoglobin in the healthy and pathological tissues. However, the error of the tumor margin evaluation with a single-fiber scheme heavily depends on the distance between the fiber and the surface of the object due to the averaging of the diffusely reflected light from the area seen by fiber’s aperture during measurement. Yet, even for the DRS-response detected at a distance of 10 mm from the surface, the error of the evaluation of the tumor margin location is not greater than 2 mm (Figure 2) and could be even lower under measurement conditions in water or saline (cystoscopy-assisted bladder surgeries are performed with a bladder filled with saline), where the numerical aperture of the fiber is lower as the refractive index of the solution is closer to the value of the refractive index of the fiber materials.

In the case of the assessment of the inhomogeneous optical phantom where the depth of the tumor-modeling heterogeneity changed (Figure 3), for typical differences between the absorption coefficients observed for healthy and tumor tissues, differences in the tumor depth (or thickness of tumor) down to 0.2 mm could be determined. This fact can be helpful for the objective assessment of the boundaries of a thin tumor, for example, in the case of a “creeping tumor”. However, in the case of unknown absorption coefficients of healthy and tumor tissues, as well as in the case of imaging of a large tissue area, a single-fiber scheme is inferior to imaging techniques, namely, SFDI.

Although the model used to estimate the absorption and scattering coefficients in the single-layer homogeneously absorbing and scattering media did not perform well in the case of the phantom with heterogeneous distribution of the chromophores presented in Figure 4a, qualitatively, μ_a_ and μ_s_’ maps (Figure 4c,d) match the distribution of the chromophores in the investigated sample.

The results obtained for diffuse reflectance coefficients and apparent optical densities (estimated as OD = −ln(*R*)) at different spatial frequencies allowed us to experimentally verify the dependency of the light penetration depth on the value of the spatial frequency (Figure 5c). We observed that the lower the frequency, the higher the penetration depth and the greater is the optical density contrast between normal and tumor tissue sites for the tumor located on some depth in optical phantom. The ratio of optical densities at 0.0 and 0.05 mm^−1^ frequencies allowed us to build an estimator of the tumor depth based solely on optical descriptors (Figure 5d). Thus, with the help of structured light, it is possible to carry out tomography of tissues, which gives additional information regarding chromophore distribution in comparison with the information provided with other imaging techniques, such as white light cystoscopy or narrow-band imaging; however, the exact solution for inhomogeneously distributed chromophores with arbitrary distribution geometry and unknown absorption coefficients has yet to be obtained.

It should also be noted that the use of structured light is technically complicated because, in order to illuminate the surface non-uniformly through an endoscope, a costly imaging fiber bundle composed of a large number of fibers is required, and for its practical applicability, it is necessary for the advantages of the SFDI method to “outweigh” the disadvantages associated with implementation difficulties.

## Data Availability

The data presented in this study are available on request from the corresponding author.

## References

[1] Baltussen E.J.M., Snæbjörnsson P., de Koning S.G.B., Sterenborg H.J.C.M., Aalbers A.G.J., Kok N., Beets G.L., Hendriks B.H.W., Kuhlmann K.F.D., Ruers T.J.M. (2017). Diffuse Reflectance Spectroscopy as a Tool for Real-Time Tissue Assessment during Colorectal Cancer Surgery. J. Biomed. Opt..

[2] Amiri S.A., Van Gent C.M., Dankelman J., Hendriks B.H.W. (2020). Intraoperative Tumor Margin Assessment Using Diffuse Reflectance Spectroscopy: The Effect of Electrosurgery on Tissue Discrimination Using Ex Vivo Animal Tissue Models. Biomed. Opt. Express.

[3] Santos I.P., Barroso E.M., Schut T.C.B., Caspers P.J., van Lanschot C.G.F., Choi D.-H., Van Der Kamp M.F., Smits R.W.H., Van Doorn R., Verdijk R.M. (2017). Raman Spectroscopy for Cancer Detection and Cancer Surgery Guidance: Translation to the Clinics. Analyst.

[4] Stenzl A., Penkoff H., Dajc-Sommerer E., Zumbraegel A., Hoeltl L., Scholz M., Riedl C., Bugelnig J., Hobisch A., Burger M. (2011). Detection and Clinical Outcome of Urinary Bladder Cancer with 5-aminolevulinic Acid-induced Fluorescence Cystoscopy: A Multicenter Randomized, Double-blind, Placebo-controlled Trial. Cancer.

[5] Poulon F., Pallud J., Varlet P., Zanello M., Chretien F., Dezamis E., Abi-Lahoud G., Nataf F., Turak B., Devaux B. (2018). Real-Time Brain Tumor Imaging with Endogenous Fluorophores: A Diagnosis Proof-of-Concept Study on Fresh Human Samples. Sci. Rep..

[6] Unger J., Hebisch C., Phipps J.E., Lagarto J.L., Kim H., Darrow M.A., Bold R.J., Marcu L. (2020). Real-Time Diagnosis and Visualization of Tumor Margins in Excised Breast Specimens Using Fluorescence Lifetime Imaging and Machine Learning. Biomed. Opt. Express.

[7] Wang M., Tang F., Pan X., Yao L., Wang X., Jing Y., Ma J., Wang G., Mi L. (2017). Rapid Diagnosis and Intraoperative Margin Assessment of Human Lung Cancer with Fluorescence Lifetime Imaging Microscopy. BBA Clin..

[8] Nguyen F.T., Zysk A.M., Chaney E.J., Kotynek J.G., Oliphant U.J., Bellafiore F.J., Rowland K.M., Johnson P.A., Boppart S.A. (2009). Intraoperative Evaluation of Breast Tumor Margins with Optical Coherence Tomography. Cancer Res..

[9] Hamdoon Z., Jerjes W., McKenzie G., Jay A., Hopper C. (2016). Optical Coherence Tomography in the Assessment of Oral Squamous Cell Carcinoma Resection Margins. Photodiagnosis Photodyn. Ther..

[10] Taruttis A., van Dam G.M., Ntziachristos V. (2015). Mesoscopic and Macroscopic Optoacoustic Imaging of Cancer. Cancer Res..

[11] Gavdush A.A., Chernomyrdin N.V., Malakhov K.M., Beshplav S.-I.T., Dolganova I.N., Kosyrkova A.V., Nikitin P.V., Musina G.R., Katyba G.M., Reshetov I.V. (2019). Terahertz Spectroscopy of Gelatin-Embedded Human Brain Gliomas of Different Grades: A Road toward Intraoperative THz Diagnosis. J. Biomed. Opt..

[12] Musina G.R., Nikitin P.V., Chernomyrdin N.V., Dolganova I.N., Gavdush A.A., Komandin G.A., Ponomarev D.S., Potapov A.A., Reshetov I.V., Tuchin V.V. (2020). Prospects of Terahertz Technology in Diagnosis of Human Brain Tumors–A Review. J. Biomed. Photonics Eng..

[13] Herr H.W., Donat S.M. (2008). A Comparison of White-light Cystoscopy and Narrow-band Imaging Cystoscopy to Detect Bladder Tumour Recurrences. BJU Int..

[14] Fus Ł.P., Górnicka B. (2016). Role of Angiogenesis in Urothelial Bladder Carcinoma. Cent. Eur. J. Urol..

[15] Zheng C., Lv Y., Zhong Q., Wang R., Jiang Q. (2012). Narrow Band Imaging Diagnosis of Bladder Cancer: Systematic Review and Meta-analysis. BJU Int..

[16] Cauberg E.C.C., Kloen S., Visser M., de la Rosette J.J., Babjuk M., Soukup V., Pesl M., Duskova J., de Reijke T.M. (2010). Narrow Band Imaging Cystoscopy Improves the Detection of Non–Muscle-Invasive Bladder Cancer. Urology.

[17] Cuccia D.J., Bevilacqua F., Durkin A.J., Tromberg B.J. (2005). Modulated Imaging: Quantitative Analysis and Tomography of Turbid Media in the Spatial-Frequency Domain. Opt. Lett..

[18] Cuccia D.J., Bevilacqua F.P., Durkin A.J., Ayers F.R., Tromberg B.J. (2009). Quantitation and Mapping of Tissue Optical Properties Using Modulated Imaging. J. Biomed. Opt..

[19] Gioux S., Mazhar A., Cuccia D.J. (2019). Spatial Frequency Domain Imaging in 2019: Principles, Applications, and Perspectives. J. Biomed. Opt..

[20] Konecky S.D., Mazhar A., Cuccia D., Durkin A.J., Schotland J.C., Tromberg B.J. (2009). Quantitative Optical Tomography of Sub-Surface Heterogeneities Using Spatially Modulated Structured Light. Opt. Express.

[21] Angelo J.P., van de Giessen M., Gioux S. (2017). Real-Time Endoscopic Optical Properties Imaging. Biomed. Opt. Express.

[22] Chen M.T., Papadakis M., Durr N.J. (2021). Speckle Illumination SFDI for Projector-Free Optical Property Mapping. Opt. Lett..

[23] Lurie K.L., Smith G.T., Khan S.A., Liao J.C., Bowden A.K. (2014). Three-Dimensional, Distendable Bladder Phantom for Optical Coherence Tomography and White Light Cystoscopy. J. Biomed. Opt..

[24] Ayers F., Grant A., Kuo D., Cuccia D.J., Durkin A.J. (2008). Fabrication and Characterization of Silicone-Based Tissue Phantoms with Tunable Optical Properties in the Visible and near Infrared Domain. Design and Performance Validation of Phantoms Used in Conjunction with Optical Measurements of Tissue.

[25] Liu G., Huang K., Jia Q., Liu S., Shen S., Li J., Dong E., Lemaillet P., Allen D.W., Xu R.X. (2018). Fabrication of a Multilayer Tissue-Mimicking Phantom with Tunable Optical Properties to Simulate Vascular Oxygenation and Perfusion for Optical Imaging Technology. Appl. Opt..

[26] Cheong W.-F., Prahl S.A., Welch A.J. (1990). A Review of the Optical Properties of Biological Tissues. IEEE J. Quantum Electron..

